# *Fasciola hepatica*: comparative metacercarial productions in experimentally-infected *Galba truncatula* and *Pseudosuccinea columella*

**DOI:** 10.1051/parasite/2015015

**Published:** 2015-04-23

**Authors:** Philippe Vignoles, Gilles Dreyfuss, Daniel Rondelaud

**Affiliations:** 1 INSERM 1094, Faculties of Medicine and Pharmacy 87025 Limoges France

**Keywords:** Experimental infections, *Fasciola hepatica*, *Galba truncatula*, Metacercaria, *Pseudosuccinea columella*

## Abstract

As large numbers of metacercariae of *Fasciola hepatica* are necessary for research, experimental infections of *Galba truncatula* and *Pseudosuccinea columella* with this digenean were carried out to determine the better intermediate host for metacercarial production and, consequently, the most profitable snail for decreasing the cost price of these larvae. Pre-adult snails (4 mm in shell height) originating from two populations per lymnaeid species were individually exposed to two or five miracidia, raised at 23 °C and followed for cercarial shedding up to their death. Compared to values noted in *G. truncatula*, the survival of *P. columella* on day 30 post-exposure was significantly greater, while the prevalence of *F. hepatica* infection was significantly lower. In the four *P. columella* groups, metacercarial production was significantly greater than that noted in the four groups of *G. truncatula* (347–453 per cercariae-shedding snail versus 163–275, respectively). Apart from one population of *G. truncatula*, the use of five miracidia per snail at exposure significantly increased the prevalence of *F. hepatica* in *P. columella* and the other population of *G. truncatula*, whereas it did not have any clear effect on the mean number of metacercariae. The use of *P. columella* for experimental infections with *F. hepatica* resulted in significantly higher metacercarial production than that noted with *G. truncatula*, in spite of a lower prevalence for the former lymnaeid. This finding allows for a significant decrease in the cost price of these larvae for commercial production.

## Abstract (fr)

Comme des quantités importantes de métacercaires de *Fasciola hepatica* sont nécessaires à des fins de recherche, des infestations expérimentales de *Galba truncatula* et de *Pseudosuccinea columella* avec ce Digène ont été réalisées dans ce but afin de déterminer quel est le meilleur hôte intermédiaire pour la production métacercarienne et, par suite, quel est le mollusque le plus rentable pour diminuer le prix de revient de ces larves. Des mollusques préadultes (4 mm de hauteur) provenant de deux populations pour chaque espèce de limnée ont été soumis individuellement à deux ou cinq miracidiums avant d’être élevés à 23° C et suivis pour les émissions cercariennes jusqu’à leur mort. Par rapport aux valeurs notées chez *G. truncatula*, la survie de *P. columella* au 30^ème^ jour post-exposition est significativement plus élevée alors que la prévalence de l’infestation par *F. hepatica* est significativement plus faible. Dans les quatre groupes de *P. columella*, la production métacercarienne est significativement plus importante que celle notée dans les quatre groupes de *G. truncatula* (347–453 par mollusque émettant des cercaires au lieu de 163–275). À l’exception d’une population de *G. truncatula*, l’emploi de cinq miracidiums par mollusque lors de l’exposition se traduit par une augmentation significative de la prévalence de *F. hepatica* chez *P. columella* et l’autre population de *G. truncatula*, alors qu’il n’y a pas d’effet net sur la production métacercarienne. L’utilisation de *P. columella* pour des infestations expérimentales avec *F. hepatica* permet d’avoir une production métacercarienne plus importante que celle fournie par *G. truncatula*, malgré une prévalence plus faible chez la première limnée. Ce résultat permet de diminuer de manière significative le prix de revient de ces larves pour la production commerciale.

## Introduction

Large numbers of metacercariae of *Fasciola hepatica* Linnaeus, 1758 [10] are necessary for research to follow qualitative and quantitative variations of morphological and/or biochemical parameters in experimentally-infected animals, or to study the effectiveness of new anthelminthic agents against fascioliasis in naturally-infected ruminants. To produce these metacercariae, it is necessary to raise the intermediate host, i.e. a freshwater gastropod belonging to the family Lymnaeidae, under laboratory conditions. In Western Europe, the snail *Galba truncatula* O.F. Müller, 1774 [11] was used in most cases (see review by Rondelaud and Barthe [16]). In the New World, most metacercariae productions were carried out using *Pseudosuccinea columella* Say, 1817 [6, 24, 25].

As cost prices for metacercarial production are dependent on the method used to raise infected snails [17], the choice of the lymnaeid species is of a great interest. Indeed, several specific techniques have been proposed in the past to raise *G. truncatula* because of its amphibious living environment [7, 9, 13, 14, 17, 19, 21, 26]. Among them, the use of 14 cm Petri dishes with dried lettuce and dead grass as food for snails and live spring moss for spring water oxygenation gave the best results for survival of infected snails [19]. In contrast, the breeding of the more aquatic *P. columella* in aquaria or in Petri dishes with fresh lettuce for snail food seems to be easier [1, 2, 4, 8, 15, 27]. In view of these findings, the following two questions arose: was the use of *P. columella* easier and more profitable to produce *F. hepatica* metacercariae? Did an increase of the miracidial dose for each snail at exposure have an effect on metacercarial production? To answer these two questions, experimental infections of pre-adult *P. columella* with *F. hepatica* were carried out using two or five miracidia per snail at exposure. Controls were constituted by pre-adult *G. truncatula* infected according to the same protocol.

## Materials and methods

### Snails and parasite

The first population of *P. columella* originated from Egypt and was from a water body (29°20′2.77″ N, 31°12′17.83″ E) at Al-Wasta, governorate of Beni Suef. The other was found at two French sites (44°23′27.31″ N, 0°32′2.43″ E and 44°23′31.18″ N, 0°29′59.30″ E) located near Castelmoron along the banks of the Lot River, department of Lot. Previous experimental infections of these two snail populations with cattle-derived miracidia of *F. hepatica* have demonstrated the high susceptibility (prevalence 42% to 60%) of these snails to this digenean [4, 5]. Adult snails, measuring 10–15 mm in height, were collected in March 2013 from the first population and in September–October 2013 from the other. They were transported to the laboratory and placed in 10 L covered aquaria with five snails per litre of permanently oxygenated spring water. These aquaria were subjected to constant conditions: temperature 23° ± 1 °C; light/dark period 12 h/12 h. Dissolved calcium concentration in spring water was 35 mg/L. Snails were fed on pesticide-free fresh lettuce leaves *ad libitum* and spring water in aquaria was changed weekly. Egg masses laid by these adult snails were collected and placed into small rearing aquaria. Newly hatched snails were fed on finely powdered lettuce and those that attained 4 ± 0.1 mm in shell height were used. For each *P. columella* population, a total of 200 snails were subjected to experimental infections.

The two populations of *G. truncatula* originated from central France and were from the communes of Migné (46°40′27″ N, 1°21′21″ E) and Thenay (46°37′23″ N, 1°26′2″ E), department of Indre. Their habitats were located on clay (favourable for snail growth) so that the upper shell height of adults was 11–12 mm. These populations were highly susceptible to experimental infection with *F. hepatica* (prevalence > 60%), as demonstrated by our team in previous experiments [22, 23]. Two hundred pre-adults, measuring 4 ± 0.1 mm in shell height and belonging to the overwintering (Migné) or the spring (Thenay) generation, were collected from each population in February (Migné) and April 2014 (Thenay). They were kept in the laboratory at 20 °C for 48 h for temperature acclimatization before being exposed to miracidia.

Eggs of *F. hepatica* were collected from the gall bladders of heavily infected cattle at the slaughterhouse of Limoges, department of Haute Vienne (central France). They were washed several times with spring water and were incubated at 20 °C for 20 days in the dark in order to obtain miracidia [12].

### Experimental protocol

Two experiments were carried out using eight groups of 100 pre-adults each. The first experiment was performed from February to April 2014 with two groups of *G. truncatula* (Migné) and two of *P. columella* (Beni Suef). Snails from two groups (one of *G. truncatula* and the other of *P. columella*) were individually exposed to bimiracidial infections. The protocol was the same for the other two groups but with five miracidia per snail. The second experiment was performed from April to June 2014 in order to verify the results from the first experiment and was carried out using two groups of *G. truncatula* (Thenay) and two of *P. columella* (Castelmoron). Snails were exposed to *F. hepatica* miracidia according to the protocol used in the first experiment. All exposures were performed for 4 h at 23 °C in 35 mm Petri dishes, each recipient containing 3.5 mL spring water. The four groups of *G. truncatula* were then raised for 30 days in 14 cm Petri dishes with 10 snails and 60 mL spring water per recipient. In each dish, small pieces of pesticide-free dried lettuce and dead *Molinia caerulea* leaf were placed, while several stems of live spring moss (*Fontinalis* sp.) ensured oxygenation of the water layer. Water and grass or lettuce leaves, if necessary, were changed daily, while cleaning of Petri dishes was done each week [19]. In contrast, the four *P. columella* groups were raised in covered, aerated 5 L aquaria. Snails were fed on pesticide-free fresh lettuce *ad libitum* and spring water in aquaria was changed weekly [4]. Petri dishes and aquaria were placed at 23° ± 1 °C in the same air-conditioned room as parent *P. columella*.

On day 30 post-exposure (p.e.), each surviving snail from the eight groups was put in a 50 mm Petri dish with 10 mL of spring water. In each dish with *G. truncatula*, small pieces of dried lettuce, dead grass and live spring moss were placed, while pieces of fresh lettuce and several spring moss stems were used for each recipient with *P. columella.* Petri dishes were then placed at 23° ± 1 °C as parent snails. Spring water and food were changed, if necessary, every day until snail death. When the first cercarial shedding occurred, surviving snails were subjected to a thermal shock every three days by placing their Petri dishes at 10°–13 °C for 3 h to stimulate cercarial exit [20, 29]. After their emergence, cercariae were counted and removed from Petri dishes. At the death of each infected snail, its shell was measured using callipers.

### Data analysis

The first two parameters were snail survival on day 30 p.e. and the prevalence of *F. hepatica* infection calculated using the ratio: number of cercariae-shedding (CS) snails/number of surviving snails on day 30 p.e. A χ^2^ test was used to compare the differences between snail survival and prevalence rates. The shell height of CS snails at their death, the lengths of the prepatent and patent periods, and the total number of metacercariae were also considered. Individual values recorded for the last four parameters were averaged and standard deviations were established for each snail group. Normality of these last values was analysed using Shapiro-Wilk test [25]. According to results given by this test, one-way analysis of variance (ANOVA), Student’s *t* test or Kruskal-Wallis test was used to establish levels of significance. All the statistical analyses were performed using Statview 5.0 software (SAS Institute Inc., Cary, NC, USA).

As the aim of the present study was to determine the better lymnaeid species for metacercarial production of *F. hepatica*, the statistical tests were only used to compare the differences between values noted for both lymnaeid species or for miracidial doses used at exposure (two or five miracidia/snail). The differences between values recorded for the two snail populations of each lymnaeid were not considered here.

As the cost price of *F. hepatica* metacercariae was dependent on the method used for breeding the snail host [17], it was interesting to determine whether the method using pre-adult *P. columella* resulted in a decrease in this price. The maintenance cost for 100 snails took into account (i) the time spent by a technician for snail exposure to miracidia, the surveillance of breeding recipients, the count of metacercariae and their transfer to Eppendorf tubes, and (ii) the purchase price of consumables and lettuce. The cost price of 100 metacercariae was calculated using the ratio: total cost of maintenance for 100 snails at miracidial exposure/(total number of metacercariae encysted on dish walls and bottoms/number of snails at miracidial exposure). This cost price did not take into account the infrastructure required for commercial production of these larvae.

## Results

Compared to *G. truncatula* subjected to bimiracidial infections (Table 1), the survival of *P. columella* on day 30 p.e. was significantly higher (χ^2^ = 27.79, *p* < 0.001), while the prevalence of *F. hepatica* infection was significantly lower (χ^2^ = 27.96, *p* < 0.001) and the shell height of CS snails significantly greater (*H* = 25.53, *p* < 0.001). The prepatent period was significantly longer (*t* = 3.41, *p* < 0.001) in *P. columella* than in the other species. In contrast, no significant difference between the lengths of patent periods was noted. Lastly, the mean numbers of metacercariae were significantly higher (*H* = 19.64, *p* < 0.01) in the two groups of *P. columella* than in *G. truncatula*. The highest totals of metacercariae were noted for *P. columella*, with 19 snails (out of 36 in the Beni Suef group) and 14 (out of 29 in the Castelmoron group) shedding more than 500 larvae per individual (data not shown).

**Table 1.** Snail survival on day 30 post-exposure, prevalence of *Fasciola hepatica* infection, shell height of cercariae-shedding (CS) snails at their death, lengths of prepatent and patent periods, and number of metacercariae in four groups of pre-adults subjected to individual bimiracidial exposures and raised at 23 °C.

Snail species	*Pseudosuccinea columella*			*Galba truncatula* (controls)		
Population	Beni Suef	Castelmoron	Total	Migné	Thenay	Total
Number of snails						
At exposure	100	100	200	100	100	200
Day 30 (%)	94 (94.0)	87 (87.0)	181 (85.5)	65 (65.0)	74 (74.0)	139 (69.5)
Number of CS snails	36	29	65	40	51	91
Prevalence (%)	38.2	33.3	35.9	61.5	68.9	65.4
Shell height of CS snails (mm)*	10.2 ± 1.7	11.2 ± 1.4	10.6 ± 1.6	6.3 ± 1.0	6.5 ± 0.9	6.4 ± 0.9
Lengths (days)*	52.5 ± 8.8	49.8 ± 6.1	51.3 ± 7.6	42.1 ± 3.7	39.5 ± 4.5	40.6 ± 4.2
Prepatent period						
Patent period	31.6 ± 12.4	27.3 ± 8.6	29.6 ± 10.7	21.3 ± 5.8	25.7 ± 8.2	23.7 ± 7.2
Metacercariae*						
Total	14,087	10,069	24,156	6556	9554	16,110
Per CS snail	391.3 ± 125.2	347.2 ± 84.8	371.6 ± 107.1	163.9 ± 74.3	187.3 ± 80.4	177.0 ± 77.4

* Mean value ± *SD*.

In quinquemiracidial infections (Table 2), the survival of *P. columella* was significantly higher (χ^2^ = 74.90, *p* < 0.001) than that of *G. truncatula*, while the prevalence of *F. hepatica* was significantly lower (χ^2^ = 499.88, *p* < 0.001). At snail death, the shell height of CS *P. columella* was significantly higher (*H* = 23.53, *p* < 0.001) than that of *G. truncatula*. Significant differences in favour of *P. columella* were noted for the prepatent period (*t* = 2.97, *p* < 0.01), the patent period (*t* = 4.60, *p* < 0.001) and the number of metacercariae (*H* = 8.89, *p* < 0.05). The highest totals of metacercariae were also noted for *P. columella*. In the Beni Suef and Castelmoron groups, 34 snails (out of 57) and 27 (out of 43), respectively, produced more than 500 metacercariae per individual (data not shown).

**Table 2.** Snail survival on day 30 post-exposure, prevalence of *Fasciola hepatica* infection, shell height of cercariae-shedding (CS) snails at their death, lengths of prepatent and patent periods, and number of metacercariae in four groups of pre-adults subjected to individual quinquemiracidial exposures and raised at 23 °C.

Snail species	*Pseudosuccinea columella*			*Galba truncatula* (controls)		
Population	Beni Suef	Castelmoron	Total	Migné	Thenay	Total
Number of snails						
At exposure	100	100	200	100	100	200
Day 30 (%)	92 (92.0)	81 (81.0)	173 (86.5)	52 (52.0)	45 (45.0)	97 (48.5)
Number of CS snails	57	43	100	44	35	79
Prevalence (%)	61.9	53.0	57.8	84.6	77.7	81.4
Shell height of CS snails (mm)*	12.1 ± 1.9	11.3 ± 2.1	11.7 ± 2.0	6.0 ± 0.8	6.6 ± 0.9	6.3 ± 0.8
Lengths (days)*	58.2 ± 7.1	60.4 ± 5.6	59.1 ± 6.5	49.2 ± 5.3	51.4 ± 5.2	50.1 ± 5.2
Prepatent period						
Patent period	32.6 ± 9.3	37.2 ± 8.5	34.5 ± 9.0	19.6 ± 3.2	14.3 ± 7.1	17.2 ± 5.5
Metacercariae*						
Total	25,856	18,065	43,921	12,136	8687	20,823
Per CS snail	453.6 ± 127.8	420.1 ± 105.3	439.2 ± 118.1	275.8 ± 74.9	248.2 ± 114.7	263.5 ± 92.5

* Mean value ± *SD*.

The values noted in the eight snail groups were also compared to determine whether the miracidial dose used for snail exposure had any significant effect on the characteristics of *F. hepatica* infection. In *G. truncatula* from Thenay, snail survival on day 30 p.e. was significantly higher (χ^2^ = 17.44, *p* < 0.01) in the two-miracidia than in the five-miracidia group. In contrast, no significant difference in snail survival between the two- and five-miracidia groups was noted for the other three snail populations. The prevalence of *F. hepatica* infection was significantly greater in the five-miracidia groups of three populations (Migné: χ^2^ = 7.59, *p* < 0.01; Beni Suef: χ^2^ = 8.60, *p* < 0.01; Castelmoron: χ^2^ = 6.34, *p* < 0.05). In each snail population considered separately, the shell height of CS snails in the two- and five-miracidia groups did not significantly differ from each other. Similar findings were also noted for the lengths of prepatent and patent periods. In *G. truncatula* from Migné, the number of metacercariae was significantly higher (*H* = 4.74, *p* < 0.05) in the five-miracidia than in the two-miracidia groups. In contrast, in the other three snail populations, the differences between the two- and five-miracidia groups were not significant.

Table 3 shows the results from the four groups subjected to five miracidia per snail. Compared to cost prices given for *G. truncatula* (10.30 Euros for both populations used), the values reported for *P. columella* (4.60 Euros = 5.06 US dollars) were one half lower.

**Table 3.** Production costs for 100 metacercariae in *Galba truncatula* and *Pseudosuccinea columella* infected with *Fasciola hepatica* (five miracidia per snail at exposure).

Parameters	*P. columella*	*G. truncatula* (controls)
Time (hrs) spent by a technician		
Exposure of snails to miracidia and preparation of Petri dishes	4.0	4.0
Surveillance of breeding recipients for snails	9.5	15.5
Count of metacercariae every three days during the patent period	28.5	21.0
Transfer of metacercariae to Eppendorf tubes by aspiration	0.33	0.33
Total	42.33	40.83
Cost of time (Euros) spent by a technician*	969.3	935.0
Purchase price of materials used for 100 snails (Euros)		
Consumables	7.51	7.51
Lettuce	4.12	2.88
Total cost for 100 snails (Euros)	980.9	945.3
Number of snails at miracidial exposure	200	200
Number of metacercariae encysted on dish walls and bottom	25,856: Beni Suef	12,136: Migné,
	18,065: Castelmoron	8,687: Thenay
Cost price for 100 metacercariae in Euros (US dollars) per snail exposed to miracidia**	3.79 (4.16): Beni Suef	7.78 (8.55): Migné
	5.42 (5.96): Castelmoron	10.88 (11.96): Thenay
	4.60 (5.06): Both	9.33 (10.26): Both

* Cost price (estimated) for a technician: 1 h = 22.9 Euros.

** 1 Euro = 1.10 US dollars.

## Discussion

The values noted for the characteristics of *F. hepatica* infection in *G. truncatula* agreed with those recorded by our team in previous experimental infections of these two populations with the digenean [21–23]. The figures noted in the Beni Suef group were close to data reported by Dar et al. [4] for the same population of *P. columella* but infected with an Egyptian isolate of *F. hepatica* miracidia. In the same way, the values noted in the Castelmoron group also correlated well with those reported by Dreyfuss et al. (unpublished data) for the same population infected with a French isolate of miracidia. The slight differences between the Beni Suef and Castelmoron groups might be due to interpopulation variability in the susceptibility of *P. columella* to this digenean, as reported by Vázquez et al. [28] for the Cuban populations of this lymnaeid.

Even though the prevalence of *F. hepatica* infection in *P. columella* was significantly lower than that noted in *G. truncatula*, the use of the former lymnaeid for the production of *F. hepatica* metacercariae was of interest for the following three reasons: (i) the survival of *F. hepatica*-exposed *P. columella* on day 30 p.e. was greater than that of *G. truncatula*, whatever the miracidial dose used for snail infection; (ii) the mean height of CS snails at their death was greater (10.2–12.1 mm compared to 6.0–6.6 mm for *G. truncatula*); and (iii) the total metacercarial production in *P. columella* was two times higher than that of *G. truncatula* when five miracidia were used for each snail at exposure. As the breeding of *P. columella* in aquaria during the first 30 days p.e. requires less daily surveillance than maintenance of 14 cm Petri dishes with *G. truncatula*, the use of *P. columella* populations susceptible to *F. hepatica* represents a good alternative to replace *G. truncatula* as a snail host and, consequently, to enhance metacercarial production under experimental conditions.

In the four groups of *P. columella*, there was a significant increase in prevalence when the number of miracidia per snail went from two to five, whereas snail survival on day 30 p.e. did not differ significantly. In contrast, the results were less clear in controls. In the *G. truncatula* groups from Thenay, snail survival was significantly lower in the five-miracidia than in the two-miracidia groups, while prevalence did not show any significant variation. The opposite finding was noted in the Migné groups, with a significantly increased prevalence in the five-miracidia snails but without clear variation in snail survival between the two- and five-miracidia groups. These differences between *P. columella* and controls can partly be explained by the method used to calculate the prevalence, i.e. the ratio between the number of CS snails and that of surviving snails on day 30 p.e. Apart from the Migné groups, the number of metacercariae in the other groups did not significantly differ from each other when the miracidial dose increased from two to five. This last result was more surprising because a positive relationship between the number of miracidia used for each snail and that of free rediae developing within the snail body was reported by Rondelaud and Barthe [16]. Competition occurring between free rediae during their development [3, 18] induced a delay in the differentiation of intraredial cercariae and their exit from the snail so that the numbers of shed cercariae were within the same scale of values in the two- and five-miracidia infections.

Compared to cost prices given for *G. truncatula*, the values reported for *P. columella* were one half lower (Table 3). These cost prices might be even lower if surviving snails in these groups were dissected during the patent period to collect metacercariae according to the method used by Rondelaud et al. [21] for *G. truncatula*. However, the use of this last technique needs to study the viability of these metacercariae in the definitive host because these larvae comprised different cohorts of free cercariae, i.e. those that just exited from their parental rediae, free cercariae whose tegument was covered by the first secretions of their cystogenous cells, and mature cercariae ready for release in water [18].

In conclusion, the use of *P. columella* for experimental infections with *F. hepatica* resulted in significantly higher metacercarial production than that noted for *G. truncatula*, in spite of a lower prevalence for the former snail species. This finding allowed a decrease in the cost price of these larvae for commercial production.
